# Efficacy and safety of brexpiprazole in early-episode schizophrenia: post hoc analysis of clinical trials in adults and adolescents

**DOI:** 10.1192/j.eurpsy.2025.2211

**Published:** 2025-08-26

**Authors:** C. Correll, B. Pflug, Z. Zhang, A. M. Palma, P. Such

**Affiliations:** 1 Department of Psychiatry, The Zucker Hillside Hospital, Glen Oaks, NY; 2 Department of Psychiatry and Molecular Medicine, The Donald and Barbara Zucker School of Medicine at Hofstra/Northwell, Hempstead, NY, United States; 3 Department of Child and Adolescent Psychiatry, Charité-Universitätsmedizin Berlin, corporate member of Freie Universität Berlin, Humboldt-Universität zu Berlin, and Berlin Institute of Health, Berlin, Germany; 4 Otsuka Pharmaceutical Development & Commercialization Inc., Princeton, NJ, United States; 5 H. Lundbeck A/S, Valby, Denmark

## Abstract

**Introduction:**

For patients with schizophrenia, effective treatment of early episodes may improve long-term outcomes, reduce the risk of relapse, and limit functional impairment.

**Objectives:**

To evaluate the efficacy and safety of brexpiprazole versus placebo in adult and adolescent patients with early-episode schizophrenia.

**Methods:**

Data were analyzed from four Phase 3, 6-week, randomized, double-blind, placebo-controlled trials: three in adults (Clinical Trials.gov: NCT01396421 [Vector], NCT01393613 [Beacon], NCT01810380 [Lighthouse]), and one in adolescents (NCT03198078 [Study 331-10-234]). For the trials in adults, patients aged 18–65 were randomized to placebo (total N=531), brexpiprazole (total N=1,093; 0.25, 1, 2 or 4 mg/day, or 2–4 mg/day, depending on the trial), or quetiapine extended-release (N=154; active reference in one trial). For the trial in adolescents, patients aged 13–17 were randomized to placebo (N=104), brexpiprazole 2–4 mg/day (N=110), or aripiprazole (N=102; active reference). Mean baseline Positive and Negative Syndrome Scale (PANSS) Total scores indicated that adult and adolescent patients were of similar disease severity. In all four trials, the primary efficacy endpoint was change from baseline to Week 6 in PANSS Total score. In this *post hoc* analysis, early-episode schizophrenia was defined as age 13–35, and ≤5 years’ duration of illness. Data from the four trials were pooled and compared between brexpiprazole 2–4 mg/day (FDA-recommended target dose in adults and adolescents) and placebo. Efficacy outcomes were analyzed using least squares (LS) mean change from baseline (mixed model for repeated measures). Safety was also evaluated.

**Results:**

The *post hoc* early-episode schizophrenia sample comprised 292 patients treated with brexpiprazole 2–4 mg, and 190 treated with placebo (analyzed for safety), of whom 289 and 187 were analyzed for efficacy, respectively. The *post hoc* efficacy sample comprised 19.3% of the corresponding efficacy sample of the adult trials (pooled), and 98.1% of the corresponding efficacy sample of the adolescent trial. In the *post hoc* efficacy sample, mean (standard deviation) baseline age was 22.4 (6.6) in the brexpiprazole group and 20.5 (6.6) in the placebo group, and baseline PANSS Total scores were 97.9 (13.5) and 100.4 (14.2), respectively. The LS mean (standard error) change from baseline to Week 6 in PANSS Total score was -21.4 (1.1) with brexpiprazole, and -17.8 (1.4) with placebo (p=0.042). The overall incidence of treatment-emergent adverse events (TEAEs) was 50.7% with brexpiprazole, and 46.3% with placebo. The TEAE with the highest incidence in the brexpiprazole group was akathisia (6.5%; placebo, 2.1%).

**Image 1:**

**Figure:**
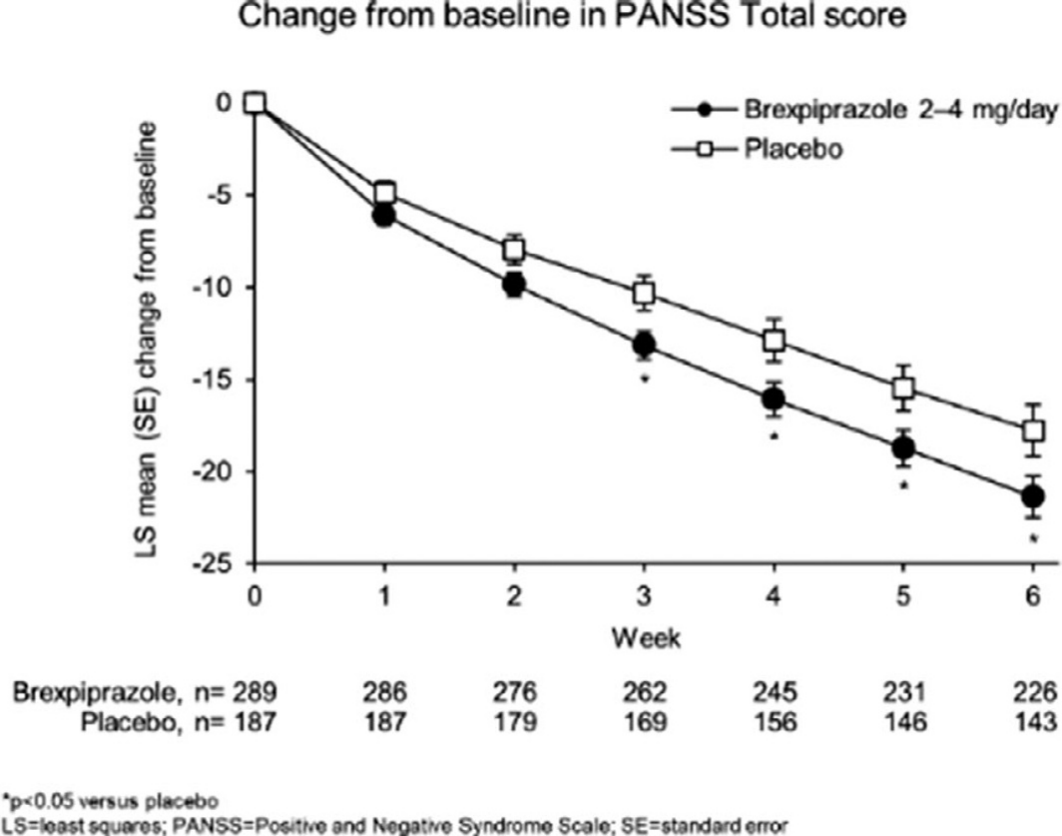

**Conclusions:**

In this *post hoc* analysis of patients with early-episode schizophrenia, brexpiprazole was associated with greater improvement in schizophrenia symptoms than placebo. No new safety observations were made.

**Disclosure of Interest:**

None Declared

